# COPD Assessment Test (CAT) score as a predictor of major depression among subjects with chronic obstructive pulmonary disease and mild hypoxemia: a case–control study

**DOI:** 10.1186/1471-2466-14-186

**Published:** 2014-11-28

**Authors:** José Laerte R Silva Júnior, Marcus Barreto Conde, Krislainy de Sousa Corrêa, Christina da Silva, Leonardo da Silva Prestes, Marcelo Fouad Rabahi

**Affiliations:** Faculdade de Medicina da Universidade Federal de Goias/UFG, Goiania, Brazil; Clínica do Aparelho Respiratório (CLARE), Av. B, n 483 setor oeste, 74110-030 Goiania, Brazil; Faculdade de Medicina de Petrópolis/FASE, Petropolis, Brazil; Instituto de Patologia Tropical e Saúde Pública da Universidade Federal de Goias/UFG, Goiania, Brazil

## Abstract

**Background:**

Depression is a common comorbidity among patients with Chronic Obstructive Pulmonary Disease (COPD) and has a significant impact on the course of the disease. The aim of this study is to determine association between COPD Assessment Test (CAT) and major depression among clinically stable out-patient COPD subjects with mild hypoxemia.

**Methods:**

Case–control study. Cases were 30 patients with major depression and controls were 30 patients without depression. Major depression was diagnosed according to the Diagnostic and Statistical Manual of Mental Disorders criteria by a psychiatric evaluation. All possible predictive variables were included in a multivariate logistic regression model to assess the association between major depression and each independent variable, while controlling for the sleep parameters.

**Results:**

CAT score >20 was associated with major depression (OR 7.88; 95% CI 1.96 - 31.7; p = 0.004).

**Conclusion:**

CAT score >20 was associated with major depression, suggesting CAT as a predictor variable of major depression among COPD patients with mild hypoxemia, and indicating that an additional specific evaluation for the presence of major depression should be done.

## Background

Chronic Obstructive Pulmonary Disease (COPD) is a medical condition with multiple comorbidities [1]. One of the most common is depression that occurs in 10 to 42% of persons with COPD and is associated with low quality of life [2, 3]. Depression is associated with increased frequency of hospital admissions, prolonged length of stay, increased number of consultations, low compliance with medical treatment and premature death [4–6].

The etiology of depression in COPD is unknown. It is supposed that a genetic predisposition is more likely to exist, followed by the environmental assaults imposed by the respiratory illness itself and the direct neuropsychiatric effects of chronic respiratory disease [7]. Little is known regarding variables associated to COPD that may predict depression [8–10].

It was hypothesized that COPD patients with symptoms of depression could report higher CAT scores (because of worse health status) compared to those without depression, as CAT scores are strongly positively correlated with St George’s Respiratory Questionnaire (SGRQ) scores [11]. Several studies addressed this issue, [11–16] and found that CAT scores are significantly associated with the presence of symptoms of depression measured by Hospital Anxiety and Depression Scale (HADS) or patient health questionnaire-9 (PHQ-9) scores, but to the best of our knowledge, there is no data evaluating the association between CAT scores and major depression diagnosed by a psychiatric evaluation. The association between depression and CAT scores could be a bias once these mentioned depression scores may indicate symptoms suggestive of depression, but not necessarily the diagnosis of a depression disease [17]. The aim of the present study is to determine the association between CAT score and major depression diagnosed according to the Diagnostic and Statistical Manual of Mental Disorders (DSM IV) criteria by a psychiatric evaluation among clinically stable out-patient COPD subjects with mild hypoxemia.

## Methods

### Design

Case–control study.

### Setting

The study was conducted at the Research Center of CLARE Outpatient Chest Clinic of Goiania (Goias, Brazil), a city localized in the middle-west of Brazil, with 1,393,575 habitants and an estimated prevalence of 16% COPD patients [18].

### Subject selection, data collection and definitions

Clinically stable COPD patients aged 40 years and over admitted to the Research Center of CLARE Out-patient Chest Clinic between April 1st and September 31st, 2013 were considered eligible and invited to take part of the study. After signing the informed consent, an instrument of data collection was applied, and a digital oximetry was performed to select only mild daytime hypoxemic patients. Subjects with oxygen saturation level < 90% (moderate to severe daytime hypoxemic patients), or ≥95% (normal oximetry), or with a FEV1/FVC >70 (not COPD by GOLD criteria) were excluded. They subsequently performed clinical evaluation, psychiatric evaluation, spirometry, polysomnography, echocardiography, arterial blood gas analysis, chest radiograph and validated questionnaires of dyspnea [19], health impairment (COPD Assessment Test - CAT) [20], socioeconomic status [21], and depression (Beck Depression Inventory - BDI, second revision) [22]. Exclusion criteria of the study were pregnancy, recent myocardial infarction (less than three months), asthma previous medical history or any other concomitant pulmonary disease, use of drugs associated with depression (lipophilic β-blockers, anabolic steroids, digoxin, levodopa, benzodiazepines and pemoline), history of cancer diagnosis, presence of renal insufficiency or performing dialysis, presence of insulin dependent diabetes, presence of resting PaO2 < 60 mmHg, presence of radiographic evidence of any significant abnormality not attributable to COPD, inability to understand or complete all questionnaires, tests and interviews. Clinical stability was defined as the absence of exacerbation [23] for the previous four weeks. Smoking status was categorized as current smokers, ex-smokers and never-smokers [24]. Major depression was diagnosed according to the Diagnostic and Statistical Manual of Mental Disorders, 4th edition (DSM IV) criteria by a psychiatric evaluation and was measured by the Beck Depression Inventory (BDI), second revision [22]. COPD subjects were classified using the GOLD criteria (chronic respiratory symptoms, history of exposure to risk factors for the disease, and post-bronchodilator FEV1/FVC ratio <70 in the spirometric evaluation) [25]. Nocturnal hypoxemia was defined as an oxygen saturation level <90% for at least 5 minutes, with a nadir oxygen saturation level of ≤85%, and was considered significant when occurring at least 30% of the total sleep time [26–29]. The polysomnography was performed using Alice 5 Diagnostic Sleep System (Murrysville, PA, USA) in a sleep laboratory. Recordings were interpreted and sleep stages were determined according to recommendations provided by the American Academy of Sleep Medicine [30].

Thirty COPD patients with depression (cases) and thirty COPD patients without depression (controls) paired by demographic characteristics were enrolled in this study.

### Sample size

n order to calculate sample size, the *t*-test was used to determine whether the COPD Assessment Test differs significantly from the COPD group with and without major depression. A previous study reported the mean and standard deviation of the CAT of out-patients COPD subjects: 24.6 ± 6.6 [31]. We intended to be able to detect a difference of 20% or more in mean CAT between groups. We calculated that would be required 28 COPD subjects in each group at a α (two-sided) = 0.05 and power = 0.80. Assuming possible losses, we chose to include two more individuals in each group totaling 60 patients.

### Statistical analysis

The results were analyzed with Stata program version 11.0 (StataCorp, Texas, USA), using *p* <0.05. Variables were described using proportions, mean and standard deviation, or median and IQR. Student’s *t*-test was used to compare two means, Mann–Whitney test was used to compare two medians, and the chi-square test or Fisher’s exact test was used for dichotomous variables. Correlation was conducted to assess the strength of linear relationships between numerical variables The unadjusted odds ratio (OR) of the association between predict variables and the outcome (major depression) variable with 95% confidence interval (CI) was calculated. All possible predictive variables were included in a multivariate logistic regression model to assess the association between major depression and each independent variable, while controlling for the sleep parameters. The cutoff value of the CAT as a depression predictor was calculated by use of a receiver operating characteristic (ROC) curve.

### Ethical aspects

This study was conducted according to good clinical practices (ICH – G6) and was approved by the Ethics Committee of the Hospital Geral of Goiania under number 198.344/2013.

## Results

**Figure 1:**
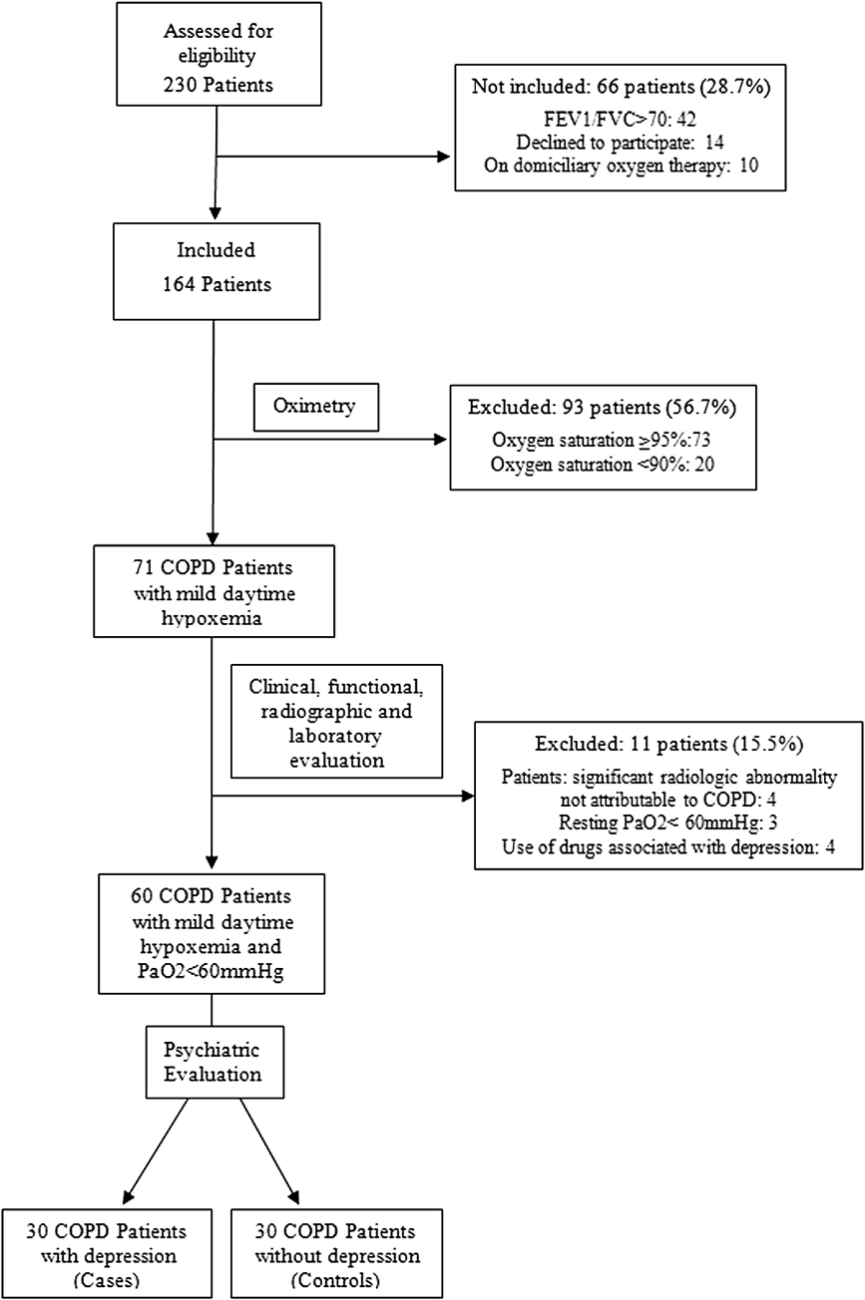
**Study design and subjects.**

Over a 6 month period, 230 patients with COPD were admitted in the Outpatient Chest Clinic and assessed for eligibility. From them, 66 patients (28.7%) were not included due to domiciliary oxygen therapy, decline to participate or FEV1/FVC ratio > 70. From the 164 included patients, 93 (56.7%) were excluded due to oxygen saturation ≥95% or <90%, 4 (2.4%) due to radiologic evidence of significant abnormalities not attributable to COPD and 3 (1.8%) resting PaO2 < 60 mmHg, and 4 (2.4%) due to use of drugs associated with depression (Figure 1).

**Figure 2:**
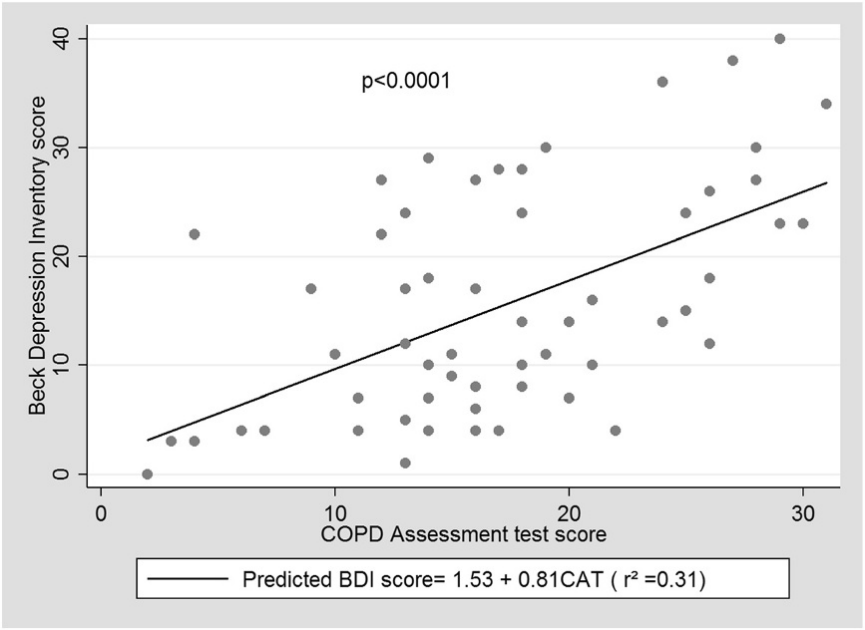
**Linear regression of Beck Depression Inventory and COPD Assessment Test scores.**

The paired variables and variables not paired of the study population are presented on Tables 1 and 2, respectively. The univariate logistic regression analysis showed that only the BDI, OR: 1.24, 95% CI 1.12-1.37, p < 0.0001; CAT, OR:1.13, 95% CI 1.04-1.24, p = 0.005; the proportion of TST of sleep stage II, OR:1.06, 95% CI 1.01-1.12, p = 0.02; and the proportion of TST of REM sleep stage OR:0.92, 95% CI 0.85-0.99, p = 0.03 predicted major depression. All other clinical (exacerbation frequency in the year prior to enrollment, gender, smoking), spirometric, gasometric and sleep variables did not predict major depression. After adjusting for TST of sleep stage II and TST of REM sleep stage, multiple logistic regression analysis revealed that only the BDI and CAT were independent predictors of major depression (Table 3). The CAT score correlated well with the BDI score (r = 0.56); *p* < 0.00001. In order to assess if CAT would significantly correlate just in the case group, we performed the same analysis in the control group and also found a statistically significant correlation (r = 0.46; *p* = 0.01). A linear regression showed that CAT significantly predicted the BDI score (Figure 2). Comparing the sensibility, specificity, positive and negative predictive value, and area under the ROC curve of each CAT cutoff, the best combination of positive predictive value and a ROC curve area without a significant loss in sensibility was CAT >20 (OR 7.88; 95% CI 1.96 - 31.7; p = 0.004).

**Table 1:** **Paired variables among cases and controls**

	All subjects	Cases	Control group	
	n = 60	n = 30	n = 30	***p***
Age, years	69.7 ± 9.0	68.8 ± 10.2	70.5 ± 7.8	0.46
Education (years)	4 (5)^†^	4 (1)^†^	4 (12)^†^	0.80
Socioeconomic score	17 (9)^†^	16 (6)^†^	19 (9)^†^	0.07

Data are presented as mean ± SD, n (%) or median (interquartile range)^†^. The Socioeconomic score was assessed by the questionnaire of the Brazilian Association of Research Companies (range 0–40).

**Table 2:** **Variables not paired among cases and controls**

		All subjects	Cases	Control group	
		n = 60	n = 30	n = 30	***p***
Male gender, n (%)		36 (60)	15 (50)	21 (70)	0.11
BMI, kg/m^2^		24.9 ± 5.0	24.5 ± 4.6	25.3 ± 5.5	0.54
Neck circumference, cm		36.4 ± 4.8	35.6 ± 4.8	37.3 ± 4.7	0.22
Systolic blood pressure, mmHg		120 (20)^†^	125 (20)^†^	120 (20)^†^	0.61
Diastolic blood pressure, mmHg		74.5 ± 8.5	74.3 ± 6.3	74.7 ± 10.4	0.88
Hypertension treatment, n (%)		34 (56.7)	18 (60)	16 (53.3)	0.60
Smoking intensity (pack years)		48.5 (33)^†^	46.5 (38.4)^†^	48.5 (32)^†^	0.83
Smoking status	Current smoker	15 (25)	9 (30)	6 (20)	
n (%)	Former smoker	40 (66.7)	17 (56.7)	23 (76.7)	0.21
	Never smoked	5 (8.3)	4 (13.3)	1 (3.3)	
COPD Classification	GOLD A	6 (10)	3 (10)	3 (10)	0.09
n (%)	GOLD B	8 (13.3)	7 (23.3)	1 (3.3)	
	GOLD C	1 (1.7)	0 (0)	1 (3.3)	
	GOLD D	45 (75)	20 (66.7)	25 (83.3)	
Pos-Bd FVC (liters)		2.52 ± 0.8	2.46 ± 0.8	2.57 ± 0.9	0.59
Pos-Bd FEV1 (liters)		1.31 ± 0.6	1.33 ± 0.6	1.29 ± 0.6	0.78
Pos-Bd FEV1/FVC (%)		75.5 ± 18.4	53 ± 12.1	49.8 ± 12.1	0.31
PaO2, mmHg		71.7 ± 8.9	71.3 ± 8.0	72.1 ± 9.7	0.73
PaCO2, mmHg		34.9 ± 5.0	34.1 ± 5.8	35.7 ± 4.2	0.24
Oxygen saturation, gasometry (%)		93.8 ± 2.0	93.9 ± 2.1	93.7 ± 1.9	0.71
LV ejection fraction (%)		69.5 (8)^†^	70 (10)^†^	69 (6)^†^	0.93
LV systolic dysfunction, n (%)		2 (3.3)	2 (6.7)	0 (0)	0.49
LV diastolic dysfunction, n (%)		43 (71.7)	22 (73.3)	21 (70)	0.77
Pulmonary hypertension , n (%)		3 (5)	1 (3.3)	2 (6.7)	0.70
Normal sPAP, n (%)		28 (46.7)	13 (43.3)	15 (50)	
Undetermined sPAP, n (%)		29 (48.3)	16 (53.4)	13 (43.3)	
Beck Depression Inventory (0–63)		15.4 ± 10.4	22.3 ± 9.6	8.6 ± 5.4	<0.0001*
COPD Assessment Test (0–40)		17.1 ± 7.1	19.8 ± 7.3	14.3 ± 5.8	0.002*

Data are presented as mean ± SD, n (%) or median (interquartile range)^†^. The Socioeconomic score was assessed by the questionnaire of the Brazilian Association of Research Companies (range 0–40). LV systolic dysfunction definition: left ventricular ejection fraction (LVEF) ≤40%. sPAP: systolic pulmonary artery pressure. Pulmonary hypertension: sPAP > 40. Undetermined sPAP: patients without tricuspid regurgitant flow.

**Table 3:** **Multivariate logistic regression analysis assessing predictive value of Beck Depression Inventory or COPD Assessment Test adjusting for sleep parameters**

Variables	Adjusted OR	CI 95%	p
Beck Depression Inventory	1.24	1.11 - 1.39	<0.0001*
Sleep stage II (% of TST)	1.07	0.99 - 1.16	0.08
REM Sleep stage (% of TST)	1.01	0.89 - 1.16	0.22
COPD Assessment Test	1.17	1.06 - 1.30	0.002*
Sleep stage II (% of TST)	1.07	1.00 - 1.15	0.06
REM Sleep stage (% of TST)	0.97	0.87 - 1.07	0.51

CI: confidence interval. OR: odds ratio. TST: total sleep time. *Statistically significant difference.

Comparing the ability to identify major depression using cutoffs of BDI II(≥14) and CAT(>20) we found that BDI-II had a specificity of 80% (95% CI 61.4%-92.3%); sensitivity of 80% (95% CI 61.4%-92.3%); positive and negative predictive values of 80% (95% CI 65.7%-89.3%), and 80% (95% CI 65.7%-89.3%), respectively; area under the ROC curve 0.80 (95% CI 0.70-0.90). CAT > 20 had a specificity of 90% (95% CI 73.5%-97.9%); sensitivity of 46.7% (95% CI 28.3%-65.7%); positive and negative predictive values of 82.4% (95% CI 59.9%-87.2%), and 61% (95% CI 51.9%-61.4%), respectively; and area under the ROC curve 0.65 (95% CI 0.54-0.76).

## Discussion

Our findings showed that CAT may be a predictor variable of major depression among COPD patients with mild hypoxemia. As CAT score is routinely used among COPD patients, value above 20 may be used as a screening test for an additional evaluation with a specific depression questionnaire. Although it is not the first time that CAT is associated with depression, it is the first manuscript demonstrating the association between CAT and major depression diagnosed by DSM IV. The majority of the studies that reported CAT-depression association [11, 13–16] were not designed specifically to investigate this issue, but addressing other primary objectives found weak to moderate correlations as a secondary finding: Hilmarsen et al. [11], using the Hospital Anxiety and Depression Scale (HADS), assessing COPD patients with symptoms of anxiety and/or depression reported a worse disease-specific health status assessed by CAT in comparison with patients without these symptoms (r = 0.35). Miyazaki et al., studying a COPD cohort designed to prospectively investigate the management of COPD comorbidities, found that the presence of depression (HADS-D ≥ 11) was significantly associated with increased scores of CAT (r^2^ = 0.15) [13]. Different studies found weak correlations (0.30 to 0.39) between CAT scores and depression scores measured by HADS [14–16]. A study designed to investigate the association between CAT and depression specifically among COPD patients found a good correlation (r = 0.63) between CAT ≥21 and depression as diagnosed by the patient health questionnaire-9 (PHQ-9) [12].

The ability of CAT to be a predictor variable of depression may be due to the capture the health status impairment of the COPD patient, as loss of functionality is a strong mediator of the development of depression in chronic illness, with an attributable risk measured at 34% [7]. This health status impairment that includes decreased mobility, inability to carry out prior occupational activities and decreased ability to participate in previously enjoyed recreational activities could act as a trigger in an inherited vulnerability to develop depression [7]. One alternate view could explain the results in the other way around: the depressive subject could make a poor self evaluation of his health status impairment causing the CAT score to increase according with the depression score. Based on this, we assessed the CAT/BDI correlation among cases and controls in order to verify if CAT would significantly correlate just in the case group. As it was shown, there was also correlation on control group, suggesting that health status impairment could be a mediator of depression in this group of patients.

The increase of the proportion of TST of sleep stage II and a reduction of the proportion of TST of REM sleep stage findings of this study are in concordance with sleep findings of older adults with depression, as they include decreased sleep efficiency, poor sleep continuity, increases in stage I and II sleep [32] and a reduction on REM sleep in the last 1/3 of the night [33].

Although there are several screening tools available to identify depressive patients (Beck Depression Inventory, second revision, Center for Epidemiologic Studies Depression Scale (CES-D), Geriatric Depression Scale (GDS), Hospital Anxiety and Depression Scale (HADS), Patient Health Questionnaire-9 (PHQ-9)) [17], none of them are currently used in the level of the primary care setting [12, 34]. CAT is a COPD-specific health status measure [20] and although it has already been demonstrated that it correlates with depression symptoms [11–16], we showed that it could not replace a specific case finding tool [22] to identify major depression. However, our findings suggests that a CAT >20 was strongly associated with major depression (OR 7.88; 95% CI 1.96 - 31.7) and could be an important tool for depression screening purposes among COPD patients with mild hypoxemia.

Our study had several limitations. We used the Student’s t test and a continuous outcome variable to estimate an appropriate number of subjects for this study design. It is known that this procedure permits a smaller sample size for a given power, but as small samples sizes are more prone to variability, this may lead to concealed bias. The choice of a dichotomous outcome, instead of a continuous one, would increase the sample size estimation and reduce variability. However, as we use a different methodology to establish the case status and our results are in concordance with others, our relatively small sample size may have not been a problem. We designed the study to address CAT as a predictor of major depression in a subgroup of COPD patients with hypoxemia. However, we exclude moderate and severe hypoxic patients in order to address in a group of hypoxemic subjects with less compromised functional status. Furthermore, the inclusion of moderate and severe hypoxic patients would increase the mean CAT score in the control group reducing the power of the sample. Consequently, a selection bias was introduced because patients of the daily ambulatory practice have different levels of hypoxemia, varying from the normoxemia to severe hypoxemia.

## Conclusions

In our sample the CAT score >20 was a predictor variable of major depression among COPD patients with mild hypoxemia, suggesting the need of an additional investigation with a specific depression questionnaire. If the evaluation by a specific instrument also indicates depression, appropriate treatment for depression and/or a referral to psychiatric assessment should be done.
